# Reproductive plasticity in the lizard *Ameiva ameiva* across distinct climatic domains in Brazil

**DOI:** 10.1098/rsos.262126

**Published:** 2026-10-28

**Authors:** Renan A. Ramalho, Selma M. de Almeida-Santos

**Affiliations:** ^1^ IBILCE-UNESP, São José do Rio Preto, São Paulo, Brazil; ^2^ Laboratório de Ecologia e Evolução, Butantan Institute, São Paulo, São Paulo, Brazil

**Keywords:** morphology, neotropical herpetofauna, reproductive cycles, seasonal variation

## Abstract

The Neotropical lizard *Ameiva ameiva* is widespread in Brazil, occurring in contrasting climatic regimes that can shape its reproductive tactics. Here, we compared reproductive traits of three populations from equatorial, tropical wet–dry and tropical equatorial domains using museum specimens to evaluate geographic variation in reproductive tactics. We examined male and female reproductive tracts macro- and microscopically to characterize individual and population-level reproductive cycles. Males produced sperm year-round, and most individuals remained in recrudescence or spermiogenesis. Females showed follicular growth and oviductal changes that tracked vitellogenic stages and seasons, revealing population-specific adjustments. Mating coincided with vitellogenesis and ovulation in all populations, and the presence of mating plugs and stored sperm may indicate that females can produce multiple clutches per reproductive season. Together, these patterns show that *A. ameiva* exhibits a continuous reproductive cycle at the individual level but a seasonal, semi-synchronous cycle at the population level. Temporal differences among populations, particularly in the timing of vitellogenesis and mating, thus demonstrate reproductive plasticity that may contribute to the species's ability to persist across diverse and changing environments.

## 1. Introduction

Reproduction is a central component of vertebrate life histories because it ensures population persistence and shapes evolutionary trajectories. Although each species displays a characteristic set of reproductive traits, environmental factors, such as temperature, precipitation, humidity and photoperiod, can modify their expression and generate variation among populations [1]. These adjustments represent reproductive tactics that reflect fine-scale responses to local conditions [1–3] and may vary within and among populations [1,4]. Despite the high diversity of Neotropical squamates, population-level comparisons of reproductive traits are limited, and most studies focus on isolated components of the reproductive system [5–7]. Consequently, we lack integrated assessments that combine macroscopic and histological evidence to understand how environmental gradients shape reproductive cycles in widely distributed species.

The lizard *Ameiva ameiva* is an excellent model for such investigations. It is widely distributed across Brazil, inhabiting xeric, seasonal and humid environments. Previous studies have reported contrasting reproductive patterns across its range, including aseasonal reproduction in the Caatinga, seasonal reproduction in Amazonian savannas, extended reproductive periods in coastal restinga and prolonged cycles in other regions [5,8–11]. These divergent scenarios suggest substantial reproductive plasticity, yet no integrated analysis has compared complete reproductive cycles using both macroscopic and microscopic data from males and females, across climatic domains.

Brazil's marked climatic and geological heterogeneity imposes different selective pressures that may influence reproductive timing and physiological adjustments, creating an ideal setting for comparative studies [5,12,13]. Understanding how populations respond to this variation is important because herpetofauna are particularly sensitive to changes in temperature, hydrological cycles and habitat structure, which can shorten activity periods and compromise reproductive success [14–16].

Here, we use *A. ameiva* as a model to investigate how populations across three Brazilian climatic domains adjust their reproductive tactics. Specifically, we aim to (i) characterize microscopic variation in male and female reproductive tracts, (ii) describe reproductive cycles at both individual and population levels, and (iii) compare the timing of vitellogenesis, spermatogenesis, mating season, sperm storage and gravidity. We expected reproductive cycles to vary among climatic domains according to local rainfall regimes, with differences in the timing, duration and synchrony of reproductive events reflecting regional variation in precipitation seasonality. By integrating macroscopic and histological data, we provide insights into the understanding of reproductive plasticity in this species and contribute to broader models of how tropical reptiles adjust their reproductive tactics in response to contrasting environmental conditions.

## 2. Material and methods

### 2.1. Study species

*Ameiva ameiva* is a medium-sized, oviparous, diurnal and terrestrial lizard [17,18] (figure 1). It forages actively in open, sun-exposed habitats, where its body temperature ranges from 33 to 40°C during activity [18]. The species occurs across tropical and subtropical South America, from Venezuela to Paraguay [19]. In Brazil, it occupies a broad range of biomes, including the Amazon Rainforest, Cerrado, Atlantic Forest, Caatinga and Pantanal [8,10,20–22].

**Figure 1.**
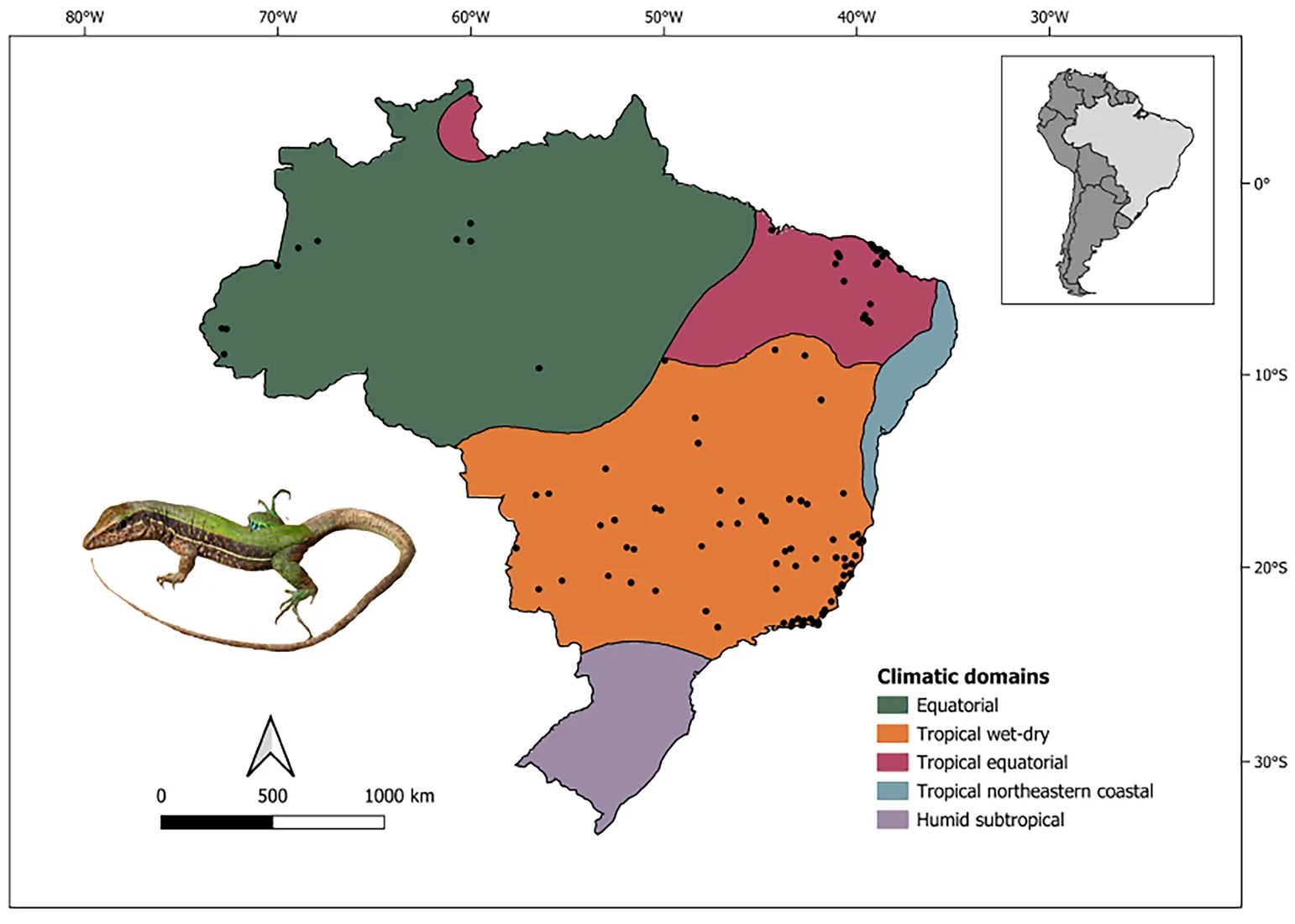
Collection localities of the *A. ameiva* specimens analysed in this study (*n* = 207). Black dots indicate all individual records included in the analyses, spanning three climatic domains sampled in this study. Photograph shows an adult *A. ameiva* (photo by Mauro Teixeira).

### 2.2. Specimens and areas

We examined 207 specimens (121 males and 86 females) from 15 Brazilian scientific collections (supplementary material, table S1). Specimens were collected between 1969 and 2022 from 87 localities assigned to three climatic domains [12,23] (figure 1):

— Equatorial: hot and humid climate of northern Brazil characterized by high annual rainfall (1600–3000 mm) and relatively low rainfall seasonality. Temperatures remain stable throughout the year (24–26°C), with a short drier period from June to November.
— Tropical wet–dry: climate characterized by pronounced rainfall seasonality across central-western and eastern–southern Brazil, with a wet season from November to March and a dry season from April to October. Annual rainfall ranges from 2000 to 3000 mm. Temperatures vary throughout the year and are closely associated with precipitation patterns.
— Tropical equatorial: warm climate covering much of northeastern Brazil, characterized by intermediate to high rainfall seasonality and considerable spatial variation in annual precipitation (620–2300 mm). Rainfall is concentrated mainly between January and May, whereas temperatures remain warm year-round (18–33°C).

These climatic domains differ primarily in the annual distribution and predictability of rainfall, allowing us to evaluate whether reproductive cycles vary according to regional precipitation regimes.

### 2.3. Macroscopic data

We measured snout–vent length (SVL) with a flexible ruler (precision = 0.5 mm) and dissected each specimen through a ventral incision from the pelvic to the pectoral girdle to expose the reproductive system [24]. In males, we measured the length, width and thickness of both testes, as well as the diameter of the ductus deferens at its caudal portion using a digital caliper (precision = 0.1 mm). In females, we recorded oviductal gross morphology (smooth or folded), the number of ovarian follicles, the length and width of the largest follicle from each ovary and the number and length of oviductal eggs.

### 2.4. Microscopic data

Samples from the right side of the reproductive tract were removed and stored in 70% ethanol for light microscopy. In males, we collected the testis, epididymis, ductus deferens and kidney; in females, the ovary, posterior infundibulum and glandular and aglandular regions of the uterus [25]. Tissues were dehydrated through an ethanol series (70–100%), cleared in xylene and embedded in paraffin [26]. Sections were cut at 5 µm with a Microm HM 340 E microtome and stained with haematoxylin and eosin. Slides were examined under an Olympus BX 51 microscope coupled with an Olympus DP73 camera.

Only specimens showing adequate tissue preservation and well-defined histological structures were included in the histomorphometric analyses. Morphometric measurements were obtained at 20× magnification using cellSens software (Olympus), based on 5–10 measurements per structure per individual. In males, we measured both the diameter of each structure and the height of its epithelium in the seminiferous tubules, epididymis, ductus deferens and sexual segment of the kidney (SSK). In females, we measured the height of the follicular, posterior infundibular and glandular uterine epithelia, as well as the diameter of the uterine glands.

We classified testicular stages into four categories (adapted from [27]): (I) quiescence—testicular inactivity; only spermatogonia and Sertoli cells lining the basal membrane; (II) recrudescence —gonadal reactivation, increased cell division, first and second meiotic phases, low sperm production; (III) spermiogenesis—peak activity, abundant sperm production and release into the seminiferous tubule lumen (i.e. spermiation); and (IV) regression—reduced germinal epithelium height, with only residual spermatozoa remaining in the lumen. We examined kidney sections to assess hypertrophy of SSK. Four SSK stages were defined (adapted from [28,29]): (I) hypertrophied SSK with few cytoplasmic granules and none in the lumen; (II) hypertrophied SSK with secretory granules concentrated in the apical cytoplasm; (III) hypertrophied SSK with numerous cytoplasmic granules but none in the lumen; and (IV) maximum hypertrophy with abundant granules in both the cytoplasm and lumen.

We classified ovarian follicles into two stages based on histological examination (adapted from [30]): (I) primary vitellogenesis—smaller follicles with a multilayered, polymorphic follicular epithelium and homogeneous central ooplasm; and (II) secondary vitellogenesis—enlarged follicles with a thinner, uniformly organized follicular epithelium and central ooplasm filled with yolk deposition vacuoles.

Male and female reproductive cycles were classified according to the framework proposed by Mathies [31]. During the examination of female reproductive tracts, the occurrence of mating plugs was recorded whenever present. Their histochemical composition was subsequently evaluated using Alcian Blue (pH 2.5) and Periodic Acid–Schiff (PAS) staining to detect acidic and neutral mucopolysaccharides, respectively.

### 2.5. Data analysis

Males were considered sexually mature when they showed turgid testes, convoluted ductus deferentia, active spermatogenesis (spermatogenic cells and mature spermatozoa in the epididymides or ducts) or a developed SSK [32,33]. Females were considered mature when they had follicles in secondary vitellogenesis, oviductal eggs, folded posterior infundibula or glandular uterus, any sign of previous reproduction (e.g. corpora lutea or distended oviducts) or sperm in the oviducts [24,25,30]. Most records of courtship and mating were obtained from published literature. For each record, we extracted the locality, date of occurrence and type of reproductive behaviour based on the behavioural description provided in the original source. Two additional records were based on direct field observations, and two mating records were inferred from the histological detection of mating plugs.

Normality and homoscedasticity were assessed using graphical inspections of residuals. Mean testicular volume per individual was calculated using the ellipsoid formula: *V* = (4/3)*abc*, where *a* = ½ length, *b* = ½ width and *c* = ½ thickness [34]. Variation between wet and dry seasons in testicular volume and ductus deferens macroscopic diameter was evaluated using analysis of covariance (ANCOVA) with SVL as a covariate, or the Mann–Whitney–Wilcoxon test when assumptions were not met and no correlation with SVL was detected (using Pearson's correlation test). Linear mixed models were used to test variation between wet and dry seasons in microscopic epithelial height and diameter of seminiferous tubules, epididymis, ductus deferens and SSK, including individual identification as a random effect. In female models, we included reproductive stage (primary or secondary vitellogenesis) as a covariate for seasonal variation in follicular, posterior infundibular, glandular uterine epithelia and uterine gland diameter. Correlations between seminiferous epithelial height and SSK epithelial height were evaluated using Pearson's correlation test. We considered differences significant at *p* < 0.05. All analyses were performed in R v. 4.3.0 (R Core Team) in RStudio v. 2023.03.1.

## 3. Results

### 3.1. Male reproductive cycle

#### 3.1.1. Testicular and ductal variation

Testicular volume and ductus deferens macroscopic diameter did not vary seasonally in any population (supplementary material, table S2). In the tropical wet–dry population, the microscopic measurements revealed significant seasonal differences: both the epididymis and the ductus deferens displayed greater histological diameter and epithelial height during the wet season than during the dry season (epididymis diameter: χ² = 4.42, *p* = 0.035; epididymis epithelium: χ² = 4.77, *p* = 0.028; ductus deferens histological diameter: χ² = 3.94, *p* = 0.049; and ductus deferens epithelium: χ² = 10.64, *p* = 0.001; table 1). By contrast, the tropical equatorial population showed a significant reduction in ductus deferens diameter during the wet season (χ² = 4.27, *p* = 0.038; table 1). No other variable changed seasonally in any population (table 1). Seminiferous epithelial height showed a weak positive correlation with SSK epithelial height in both the tropical wet–dry (*r* = 0.20, *p* < 0.001) and tropical equatorial (*r* = 0.22, *p* = 0.001) populations.

**Table 1.** Mixed-effects model results for morphometric measurements of seminiferous tubules (ST), epididymis (Ep), ductus deferens (DD) and sexual segment of the kidney (SSK) in *A. ameiva* across three climatic domains*.*

population	structure	measurement	estimate (wet season)	statistic (X²)	*p*-value
equatorial	ST	diameter	23.42	0.92	0.337
		epithelium	2.55	0.05	0.818
	Ep	diameter	13.26	0.26	0.608
		epithelium	–1.18	0.08	0.769
	DD	diameter	–1.78	< 0.01	0.961
		epithelium	–1.06	1.13	0.287
	SSK	diameter	–9.32	0.86	0.353
		epithelium	–1.40	0.16	0.688
tropical wet–dry	ST	diameter	–16.97	1.91	0.166
		epithelium	1.17	0.14	0.703
	Ep	diameter	21.42	4.42	**0.035**
		epithelium	3.11	4.77	**0.028**
	DD	diameter	22.60	3.94	**0.049**
		epithelium	2.27	10.64	**0.001**
	SSK	diameter	4.14	0.73	0.391
		epithelium	–3.14	3.40	0.065
tropical equatorial	ST	diameter	–54.24	3.26	0.070
		epithelium	–5.07	1.21	0.270
	Ep	diameter	–12.02	0.22	0.637
		epithelium	–3.70	3.30	0.068
	DD	diameter	–55.05	4.27	**0.038**
		epithelium	–2.71	2.20	0.137
	SSK	diameter	–16.10	2.73	0.097
		epithelium	–2.18	0.31	0.575

*p-values* < 0.05 are indicated in bold.

#### 3.1.2. Spermatogenic stages and sexual segment of the kidney hypertrophy

The relative frequency of spermatogenic stages varied among populations and between seasons. Quiescence was absent in all populations. In the equatorial and tropical equatorial populations, recrudescence and spermiogenesis were the predominant stages, although their relative frequencies varied between seasons (table 2). In the tropical wet–dry population, spermiogenesis predominated in both seasons (table 2). Overall, recrudescence and spermiogenesis accounted for more than 90% of males in both the wet (31.4% and 61.1%) and dry seasons (32.6% and 65.3%), whereas regression remained rare (7.4% and 1.9%, respectively).

**Table 2.** Monthly occurrence of male *A. ameiva* in each spermatogenic stage across three climatic domains.

	stages (wet season)				stages (dry season)			
population	I	II	III	IV	I	II	III	IV
equatorial	—	Dec (1) Feb (2)	Feb (1) Apr (1) May (1)	Jan (1)	—	Jul (1) Nov (1)	Jun (1) Jul (1) Aug (2) Set (1) Nov (1)	—
tropical wet–dry	—	Nov (2) Jan (3) Feb (1) Mar (4)	Nov (7) Dec (2) Jan (7) Feb (5) Mar (4)	Mar (1)	—	Apr (2) May (1) Jul (1) Aug (1) Sep (3) Oct (5)	Apr (4) May (2) Jun (2) Jul (1) Aug (3) Sep (5) Oct (5)	—
tropical equatorial	—	Feb (1) Apr (1) May (2)	Jan (1) Mar (3) Apr (1)	Mar (1) Apr (1)	—	Jul (1) Oct (1)	Jun (1) Jul (1) Aug (2) Oct (1) Dec (1)	Dec (1)

Spermatogenic stages: I—quiescence; II—recrudescence; III—spermiogenesis; IV—regression. Entries indicate the month of collection followed by the number of individuals in parentheses.

Patterns in the SSK paralleled the testicular results. Hypertrophy stages III and IV predominated in the equatorial and tropical equatorial populations, whereas stage III was most frequent in the tropical wet–dry population (table 3). Stage III was the most common stage across the sampled months, representing roughly half of the individuals in both the wet and dry seasons (54.3% and 51.8%, respectively). Stages II and IV together accounted for 43.8% of males in the wet season and 40.7% in the dry season. Stage I was rare accounting for less than 2% of individuals in the wet season and less than 10% in the dry season.

**Table 3.** Monthly occurrence of male *A. ameiva* in each stage of hypertrophy of the sexual segment of the kidney (SSK) across three climatic domains.

	stages (wet season)				stages (dry season)			
population	I	II	III	IV	I	II	III	IV
equatorial	—	May (1)	Feb (1) Mar (1)	Dec (1) Jan (1) Feb (1) Apr (1)	—	Jul (1)	Jun (1) Jul (1) Aug (1) Set (1) Nov (1)	Aug (1) Nov (1)
tropical wet–dry	Mar (1)	Nov (2) Jan (1) Feb (1) Mar (4)	Nov (5) Dec (2) Jan (7) Feb (5) Mar (2)	Nov (2) Jan (3) Mar (3)	Apr (1) May (1) Jun (1) Sep (1)	Jul (1) Sep (1) Oct (2)	Apr (2) Jun (1) Jul (2) Aug (3) Sep (5) Oct (3)	Apr (2) May (2) Sep (1) Oct (5)
tropical equatorial	—	Feb (1) Mar (1)	Jan (1) Mar (3) Apr (2) May (2)	Feb (1) Apr (1)	—	Jul (1)	Jun (1) Aug (3) Oct (2) Dec (1)	Jul (1) Aug (1) Oct (1) Dec (1)

SSK hypertrophy stages: I—hypertrophied SSK with few secretion granules in the cytoplasm and none in the lumen; II—hypertrophied SSK with secretion granules concentrated in the apical cytoplasm; III—hypertrophied SSK with abundant secretion granules in the cytoplasm but absent from the lumen; IV—maximal SSK hypertrophy, with abundant secretion granules in both the cytoplasm and the lumen. Entries indicate the month of collection followed by the number of individuals in parentheses.

#### 3.1.3. Temporal distribution of male reproductive activity

The timing of reproductive activity varied subtly among populations. Testicular recrudescence occurred year-round in the tropical wet–dry population and from February to October in the tropical equatorial population (figures 2 and 3*a*). In the equatorial population, recrudescence was recorded in February, July, November and December, with most records occurring around the transition between the dry and wet periods (figures 2 and 3*a*). Spermiogenesis persisted throughout the year in all populations (figures 2 and 3*b*), whereas regression was more frequent from December–April in the tropical equatorial population, in March in the tropical wet–dry population and in January in the equatorial population (figures 2 and 3*c*). Spermatozoa were consistently present in the epididymis and ductus deferens, and the SSK remained hypertrophied year-round in all populations (figures 2 and 3*d–f*).

**Figure 2.**
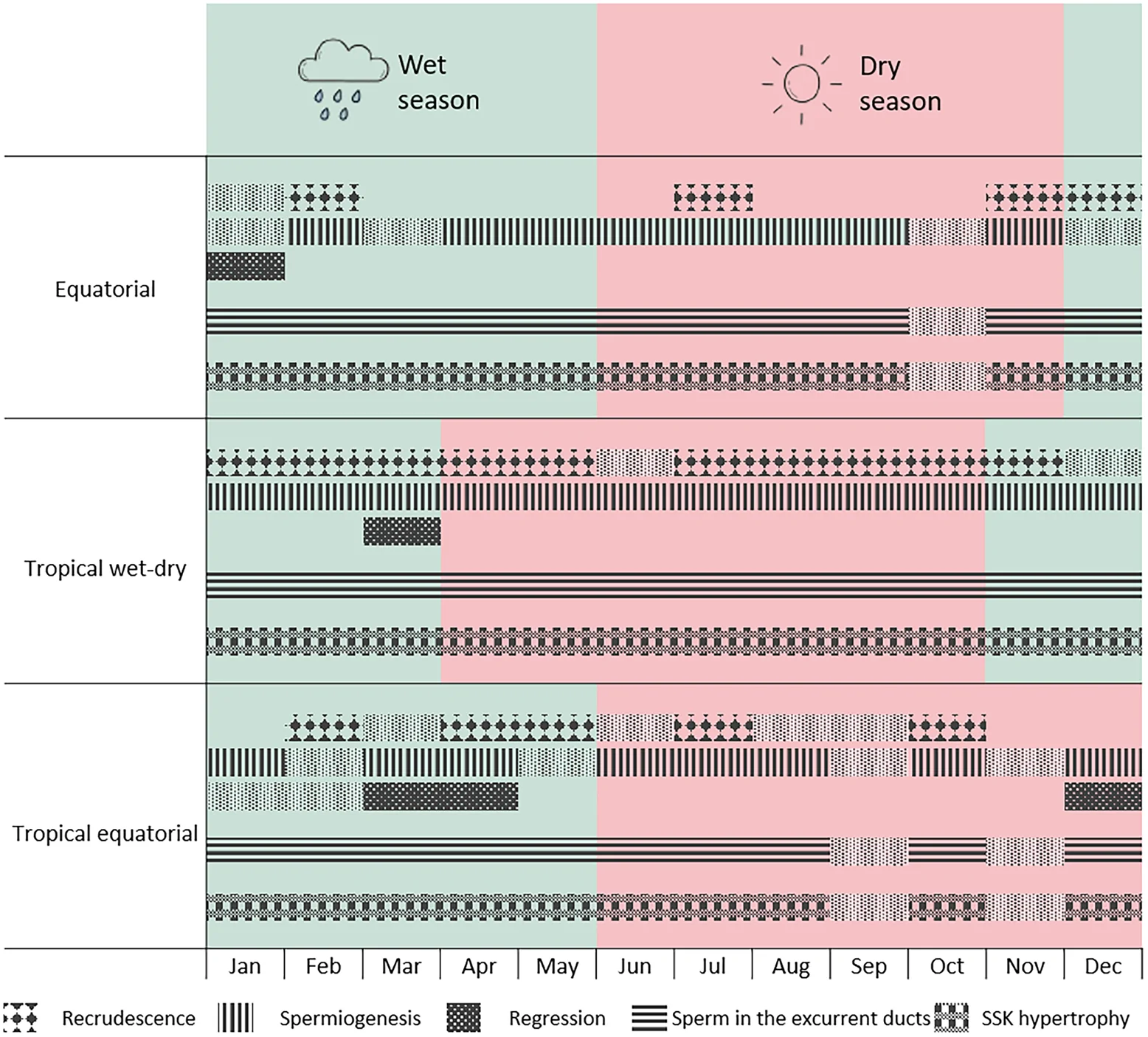
Seasonal distribution of male reproductive events in *A. ameiva* from three Brazilian climatic domains. Dotted areas denote estimated intervals inferred from incomplete monthly sampling.

**Figure 3.**
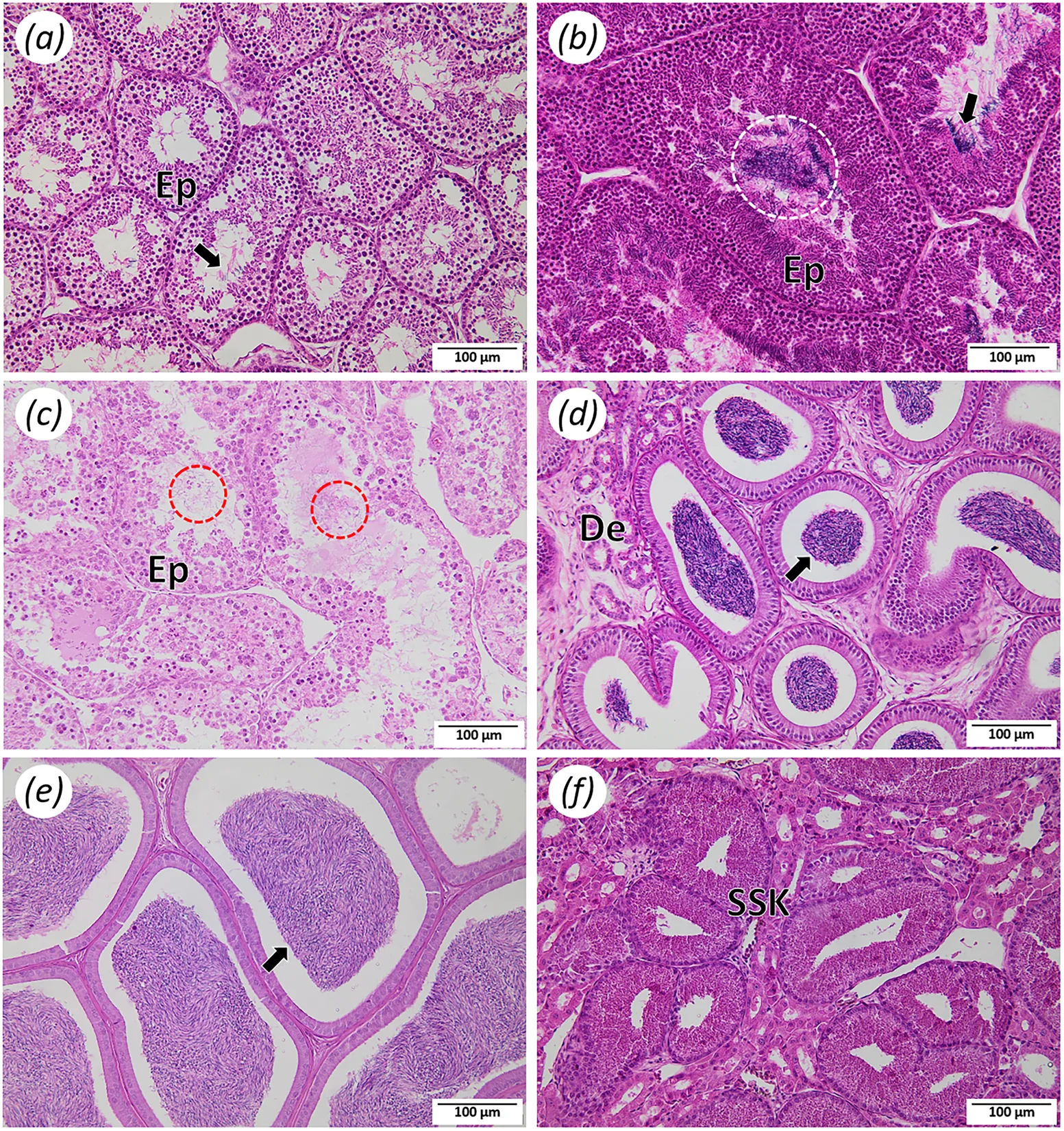
Histological sections (haematoxylin and eosin) of the male reproductive tract of *A. ameiva*. (*a*) testis in recrudescence, showing reactivation of the seminiferous epithelium with low sperm production (arrow). (*b*) Testis in spermiogenesis with abundant sperm production (arrow) and release into the seminiferous tubule lumen (dashed circle). (*c*) Testis in regression, with reduced germinal epithelium height and residual spermatozoa in the lumen (dashed circles). (*d*) Epididymis containing abundant spermatozoa (arrow). (*e*) Ductus deferens with luminal spermatozoa (arrow). (*f*) Hypertrophied SSK. Ep: seminiferous epithelium; de: ductuli efferentes.

### 3.2. Female reproductive cycle

#### 3.2.1. Follicular development

In the tropical wet–dry population, follicular epithelium height was significantly lower during the wet season than during the dry season (χ² = 4.14, *p* = 0.041; table 4). Females in secondary vitellogenesis also had thinner follicular epithelia than those in primary vitellogenesis (χ² = 49.00, *p* < 0.001; table 4). These females also showed higher epithelial height in the posterior infundibulum (χ² = 27.67, *p* < 0.001; table 4) and glandular uterus (χ² = 20.52, *p* < 0.001; table 4), as well as larger uterine gland diameter (χ² = 28.28, *p* < 0.001; table 4). In the equatorial and tropical equatorial populations, the only significant change was the reduction in follicular epithelial height in females in secondary vitellogenesis (equatorial: χ² = 10.21, *p* = 0.001; tropical equatorial: χ² = 3.91, *p* = 0.047; table 4). No other variable differed between seasons or reproductive stages ( table 4).

**Table 4.** Mixed-effects model results for morphometric measurements of follicular (EpF), posterior infundibulum (PI), uterine (EpU) epithelium and uterine gland diameter (UG) in *A. ameiva* across three climatic domains*.* RS: reproductive stage; V2: secondary vitellogenesis.

population	measurement	predictor	estimate (wet season/V2)	statistic (X²)	*p*-value
equatorial	EpF	season	–0.25	< 0.01	0.979
		RS	–32.96	10.21	**0.001**
	PI	season	1.31	1.22	0.267
		RS	1.16	0.70	0.400
	EpU	season	–2.21	2.30	0.129
		RS	–0.80	0.24	0.620
	UG	season	–5.27	1.59	0.206
		RS	5.45	1.26	0.261
tropical wet–dry	EpF	season	–12.53	4.14	**0.041**
		RS	–44.28	49.00	**< 0.001**
	PI	season	–0.52	0.19	0.655
		RS	5.73	27.67	**< 0.001**
	EpU	season	–0.77	0.33	0.560
		RS	5.55	20.52	**< 0.001**
	UG	season	0.90	0.07	0.778
		RS	15.78	28.28	**< 0.001**
tropical equatorial	EpF	season	19.61	0.46	0.494
		RS	–40.13	3.91	**0.047**
	PI	season	–0.25	0.01	0.905
		RS	2.04	0.91	0.338
	EpU	season	0.24	0.05	0.814
		RS	0.15	0.02	0.885
	UG	season	0.29	0.004	0.949
		RS	0.83	2.38	0.122

*p-values* < 0.05 are indicated in bold.

In the equatorial population, the smallest secondary vitellogenic follicle measured 2.55 mm, and all follicles ≥ 4.90 mm were in secondary vitellogenesis. Among follicles ranging from 2.55 to 4.90 mm in diameter, 80% (8/10) were in secondary vitellogenesis. In the tropical wet–dry population, the smallest secondary follicle measured 1.99 mm, and all follicles ≥ 3.83 mm had reached this stage. About 23% (3/13) of follicles between 1.99 and 3.83 mm were in secondary vitellogenesis.

#### 3.2.2. Temporal distribution of vitellogenesis and gravidity

In the equatorial population, secondary vitellogenesis started in July and peaked from January to April (figure 4*a*). Gravid females were recorded in July, August and December (figure 4*a*), and all carried concurrent vitellogenic follicles. In the tropical equatorial population, secondary vitellogenesis extended from April to October, and gravid females occurred in March, June and July (figure 4*b*). One gravid female also carried secondary follicles, and another had residual sperm in the aglandular uterus (figure 4*b,d*). In the tropical wet–dry population, secondary vitellogenesis began in January, increased through the following months and peaked in March, in the following wet season (figure 4*c*). Gravid females occurred in May, July, October, November, January and February (figure 4*c*), and eight of them also had concurrent vitellogenic follicles. Stored sperm appeared in the aglandular uterus in March and May, and in the posterior infundibulum in November (figure 4*c*,*e*,*f*). The female from May was gravid and also carried secondary follicles.

**Figure 4.**
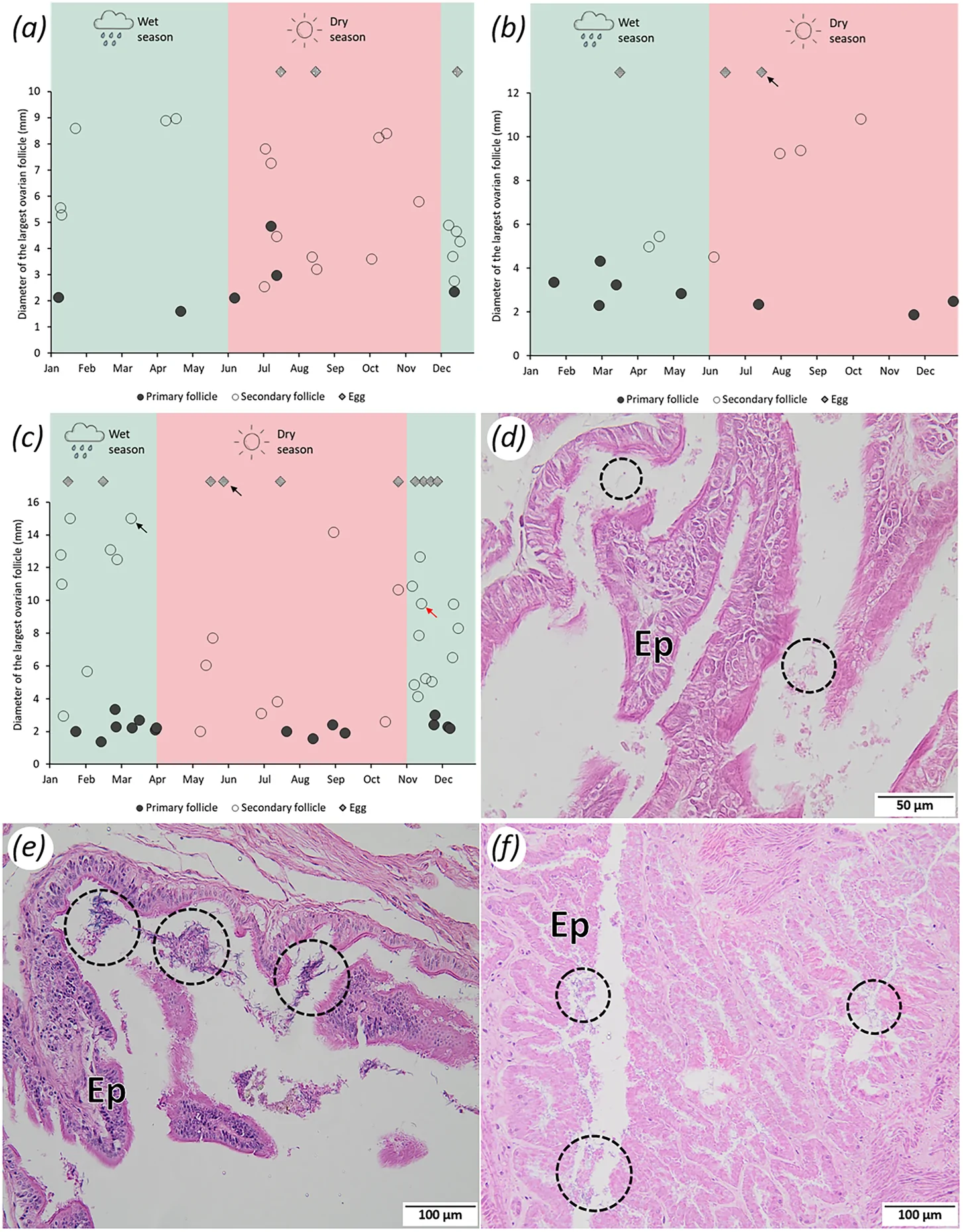
Seasonal variation in follicular development and occurrence of gravid females in *A. ameiva* across three Brazilian climatic domains: (*a*) equatorial, (*b*) tropical equatorial, and (*c*) tropical wet–dry. Panels (*d–f*) show histological sections (haematoxylin and eosin) illustrating the presence of spermatozoa (dashed circles) in the aglandular uterus of the tropical equatorial population (*d*), the tropical wet–dry population (*e*), and in the posterior infundibulum of the tropical wet–dry population (*f*). The black arrows indicate spermatozoa in the aglandular uterus, and the red arrow, in the posterior infundibulum. Ep: epithelium.

### 3.3. Mating season

Courtship and mating in the tropical wet–dry population occurred from September to October and from December to March (figure 5*a* and table 5). A mating plug was found in the aglandular uterus of a female in secondary vitellogenesis (follicle = 8.3 mm) collected in December (figure 5*b–d* and table 5). The plug consisted of an amorphous, granular matrix that filled the lumen of the caudal aglandular uterus, lacked spermatozoa and stained positive for Alcian Blue and PAS (figure 5*c,d*). In the equatorial population, one courtship event was recorded in February, and a mating plug as that described above was observed in a secondary vitellogenic female (follicle = 8.40 mm) collected in October (figure 5*e* and table 5). Conversely, in the tropical equatorial population, courtship and mating were recorded from April to September (figure 5*f* and table 5).

**Figure 5.**
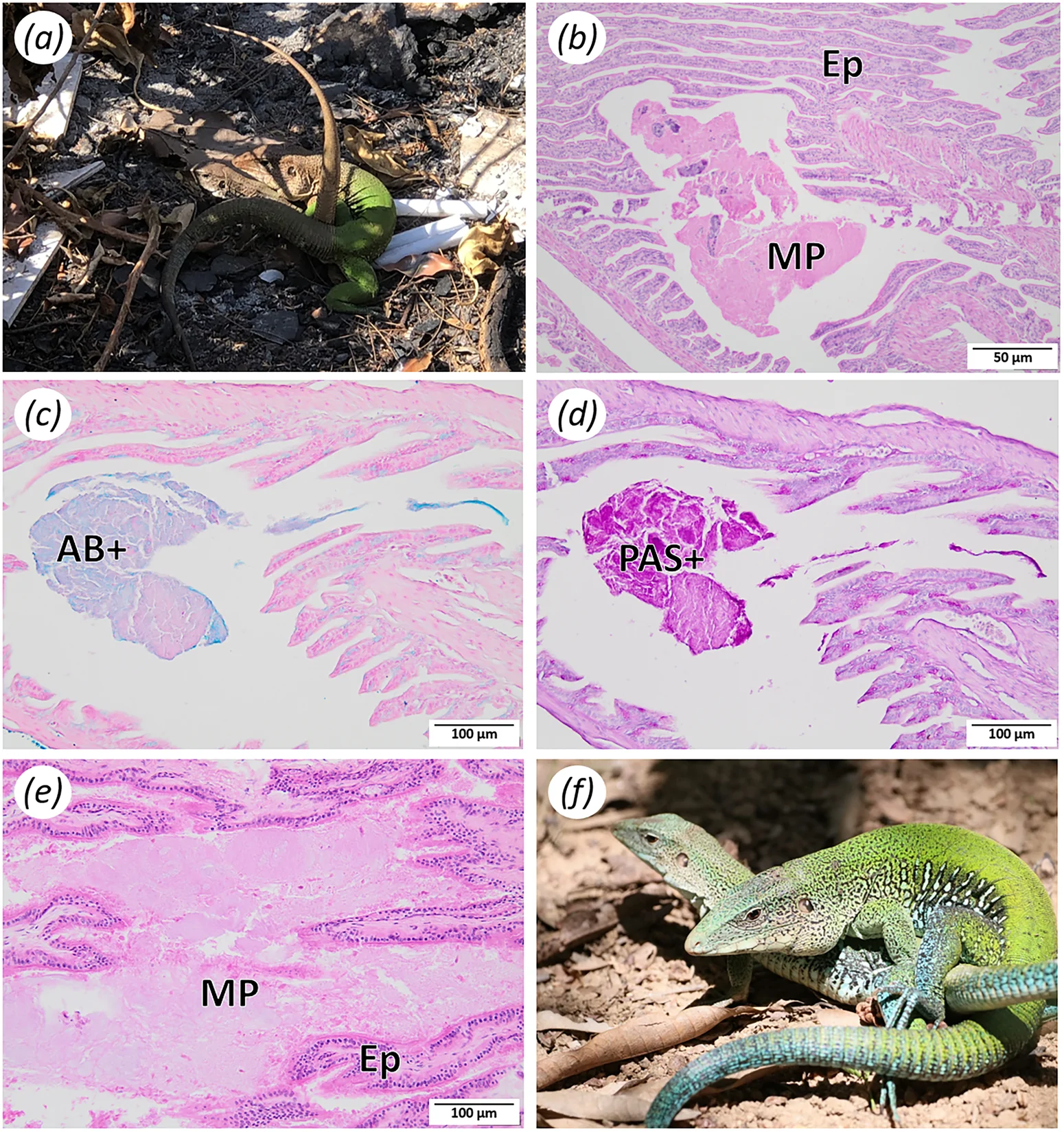
Courtship and copulation events and histological characterization of mating plugs in *A. ameiva*. (*a,f*) Courtship and copulation recorded in the tropical wet–dry population in September, in Ribeirão Preto, state of São Paulo (*a*), and in the tropical equatorial population in June, in Currais Novos, state of Rio Grande do Norte (*f*). (*b–e*) Histological sections of mating plugs (MP) located in the aglandular uterus of females. (*b*) Mating plug observed in the tropical wet–dry population stained with haematoxylin and eosin. (*c*) Alcian Blue positive reaction (AB+) indicating acidic mucopolysaccharides. (*d*) Periodic Acid–Schiff positive reaction (PAS+) indicating neutral mucopolysaccharides. (*e*) Mating plug observed in the equatorial population stained with haematoxylin and eosin. Ep: epithelium.

**Table 5.** Locality, date and type of reproductive events recorded for *A. ameiva* across three climatic domains. Events include direct *in situ* observations and histological evidence (mating plugs). Some records were obtained from the literature (superscript numbers refer to sources listed below).

locality/state	date	season	observation
**equatorial**			
Rio Xingu, Pará^1^	8 Feb 1987	wet	courtship
Manaus, Amazonas	18 Oct 2007	dry	mating plug
**tropical wet–dry**			
Serra do Cipó, Minas Gerais^2^	22 Oct 2001	dry	courtship
Ribeirão Preto, São Paulo^4^	Mar 2005	wet	mating
Inhapim, Minas Gerais^3^,^5^	12 Mar 2009	wet	courtship and mating
Vitória, Espírito Santo^4^	7 Oct 2012	dry	mating
Cariacica, Espírito Santo^4^	3 Dec 2018	wet	mating
Olímpia, São Paulo	27 Oct 2019	dry	courtship and mating
Brodowski, São Paulo^4^	10 Jan 2020	wet	courtship and mating
Ribeirão Preto, São Paulo	25 Sep 2023	dry	mating
Corumbá, Mato Grosso do Sul	17 Dec 2011	wet	mating plug
Palmeiras, Bahia^4^	17 Dec 2018	wet	mating
**tropical equatorial**			
Cantá, Roraima^4^	29 May 2019	wet	mating
Brejinho, Pernambuco^6^	30 Jun 2018	dry	mating
Brejinho, Pernambuco^6^	1 Jul 2018	dry	mating
Brejinho, Pernambuco^6^	13 Apr 2019	wet	mating
Brejinho, Pernambuco^6^	13 Apr 2019	wet	mating
Brejinho, Pernambuco^6^	30 Apr 2019	wet	mating
Brejinho, Pernambuco^6^	21 May 2020	wet	mating
Brejinho, Pernambuco^6^	28 May 2020	wet	mating
Brejinho, Pernambuco^6^	17 Jun 2020	dry	mating
Brejinho, Pernambuco^6^	6 Jul 2020	dry	mating
Brejinho, Pernambuco^6^	6 Jul 2020	dry	mating
Brejinho, Pernambuco^6^	13 Jul 2020	dry	mating
Brejinho, Pernambuco^6^	13 Jul 2020	dry	mating
Brejinho, Pernambuco^6^	2 Aug 2020	dry	mating
Brejinho, Pernambuco^6^	2 Aug 2020	dry	mating
Brejinho, Pernambuco^6^	3 Aug 2020	dry	mating
Brejinho, Pernambuco^6^	19 Aug 2020	dry	mating
Brejinho, Pernambuco^6^	9 Sep 2020	dry	mating
Brejinho, Pernambuco^6^	13 Apr 2022	wet	mating
Brejinho, Pernambuco^6^	13 Apr 2022	wet	mating
Currais Novos, Rio Grande do Norte	4 Jun 2026	dry	courtship and mating

^1^ Pianka & Vitt [35].

^2^ Manata & Nascimento [36].

^3^ Costa *et al*. [37].

^4^ Ramalho *et al*. [22].

^5^ Ramalho *et al*. [38].

^6^ J. D. de Sousa & M. N. de Carvalho Kokubum 2026, personal communication.

## 4. Discussion

We examined geographic variation in the reproductive tactics of *A. ameiva* across three Brazilian climatic domains. By combining macroscopic and histological data, we show that both sexes exhibit year-round reproductive capacity, but the timing, synchrony and intensity of the reproductive events differ among populations. These differences reveal substantial reproductive plasticity, supporting the hypothesis that this flexibility may contribute to the species's broad ecological tolerance and wide geographic distribution.

Our dataset is based on museum specimens collected over more than five decades from multiple localities across three major climatic domains. The extensive temporal and spatial coverage provides a comprehensive overview of reproductive variation across the species's geographic distribution. However, short-term climatic anomalies (e.g. El Niño events or atypical droughts), long-term climatic trends and anthropogenic environmental changes were not explicitly incorporated into our analyses. Therefore, the reproductive patterns described here should be interpreted as reflecting regional climatic regimes rather than year- or locality-specific environmental conditions.

### 4.1. Male reproductive cycle

The lack of seasonal changes in testis volume and macroscopic ductus deferens diameter across populations suggests that males maintain gonadal function throughout the year. Testis size often correlates with spermatogenic activity in *A. ameiva* [8,9,20,39], but morphometric traits alone may not reflect the underlying physiological state because hormonal fluctuations or temporary fluid accumulation can also alter organ dimensions [27,40,41]. Histology, therefore, provides a more direct assessment of reproductive activity than gross morphology. Nevertheless, preservation-related artefacts cannot be completely ruled out in museum specimens and should be considered when interpreting quantitative histological measurements.

Histological changes were detected in some populations. In the tropical wet–dry population, epididymal and ductus deferens epithelia increased in height during the wet season, which suggests subtle adjustments in sperm storage capacity at this time. By contrast, the tropical equatorial population showed a reduction in ductus deferens diameter during the wet season, supporting the idea that reproductive traits in *A. ameiva* are flexible and adjust to local climatic conditions. Despite these fluctuations, both the epididymides and ductus deferentia remained filled with sperm throughout the year in all populations, suggesting year-round reproductive capability. Future studies incorporating sperm counts and seminal volume [31,42,43] would help determine whether quantitative changes in sperm availability also occur seasonally.

 Spermatogenesis patterns were also consistent with continuous gonadal activity [31]. The absence of quiescence and the predominance of recrudescence and spermiogenesis, combined with the low frequency of regression, indicate continuous spermatogenesis. This pattern is common in tropical lizards, which alternate between recrudescence and spermiogenesis and can reproduce at higher rates than temperate climate species [27,44–46]. Similar patterns have been reported in several teiid and non-teiid genera, including *Ameiva*, *Anolis*, *Aspidoscelis*, *Cnemidophorus*, *Sceloporus* and *Tropidurus* [20,39,47–51]. Nevertheless, it is important to clarify concepts regarding the cyclical nature of male gonads in squamates, since a continuous cycle does not imply a constant rate of gamete production throughout the year. In fact, what has been observed is that there is a reduction in testicular activity during certain periods [31,43].

Patterns in the SSK matched those in the testes. The SSK remained predominantly in hypertrophy stage III throughout the year across populations. This matches the extended spermatogenic activity observed in the species and aligns with the known androgen dependence of SSK hypertrophy [52,53]. Moreover, we found a weak but positive and statistically significant correlation between SSK epithelial height and seminiferous epithelium height in two populations, supporting a functional link between these traits.

The timing of recrudescence and regression also varied among populations. In the tropical wet–dry and tropical equatorial populations, recrudescence occurred across most of the year, whereas regression seemed to concentrate in the wet season. This prolonged recrudescence suggests asynchronous individual cycles, which could keep a high proportion of males reproductively competent in the population and ensure continuous mating opportunities, particularly when reproductive females are available year-round [27,54], which is the case for *A. ameiva*.

Taken together, our results show that male *A. ameiva* maintain a continuous spermatogenic cycle at the individual level [31], characterized by a brief period of testicular regression. At the population level, male reproductive cycles are seasonal and semi-synchronous, with shifts in the timing of recrudescence and in the distribution of recrudescence and spermiogenesis across seasons. The persistent presence of spermatozoa in the excurrent ducts throughout the year in all populations suggests that males retain the capacity to fertilize females whenever mating opportunities arise.

### 4.2. Female reproductive cycle

Histology of the female reproductive tract reflected both the follicular stage and local climatic conditions. The reduction in follicular epithelial height during secondary vitellogenesis across all populations matches the expected cellular remodelling associated with yolk deposition [30]. In the tropical wet–dry population, however, increases in infundibular and uterine epithelial height, along with enlarged uterine glands, reflect oviductal preparation for ovulation, fertilization and egg development [25].

Because follicular stages were assigned using histological criteria, secondary vitellogenesis in follicles that were approximately 1 mm smaller than the thresholds derived from macroscopic criteria [8]. Therefore, follicle diameter should be interpreted as a descriptive trait associated with each histological stage rather than as a diagnostic criterion for stage assignment. This reinforces that traditional size-based criteria may overestimate the onset of vitellogenesis [24,30].

Temporal patterns of vitellogenesis varied clearly among populations. In the equatorial population, secondary vitellogenesis began during the dry season and peaked in the wet season. In the tropical wet–dry population, it started in the wet season, continued through the dry season and peaked in the following wet season. In the more xeric tropical equatorial population, vitellogenesis was more restricted, occurring mainly from the late wet season to mid-dry season. Some studies have characterized *A. ameiva* as an aseasonal or ‘extended’ breeder [5,8–11], but this interpretation often relied only on the presence or absence of vitellogenic females. When proportions are analysed, clear peaks in vitellogenesis become evident, and these peaks vary geographically. For instance, Vitt [8] reported a higher frequency of vitellogenic females during the dry season (June–August), followed by an increase in non-reproductive females in the wet season, with oviposition occurring as vitellogenesis declined. Magnusson [9] described a more concentrated wet-season cycle in the Amazonian savannas, whereas Vitt & Colli [5] documented a more extended pattern in other localities. Colli [20] found a higher proportion of reproductive females in the wet season and none in the dry season, with oviposition in the late wet and early dry seasons. Rocha [10] suggested a lack of seasonality in a coastal restinga population, although proportional data were not presented. Our findings align with these reports and expand them by providing histological confirmation of reproductive timing.

Similar inconsistencies appear in studies from Venezuela, Guyana, Ecuador and Peru. Broad surveys that recorded gravid females from February to December led to the view that reproduction was aseasonal [55–57]. However, more detailed analyses revealed a different pattern. Simmons [58] reported an increase in follicular growth from July to December, with oviposition in July and August. Duellman [59] also emphasized seasonal dynamics, noting more gravid females in the late dry and early wet seasons.

Females of *A. ameiva* can produce multiple clutches (up to three) within a reproductive season [8,11,20]. Our data confirm this pattern, as some individuals carried both vitellogenic follicles and oviductal eggs simultaneously. This ability, also reported for several other lizard species [60–64], depends on the capacity to maintain vitellogenesis during gravidity. As an active forager [8,20], *A. ameiva* has been proposed to fuel vitellogenesis primarily through recent food intake (income breeding), although previous studies have also reported reserve use (capital breeding [8]), potentially indicating energetic flexibility in this species and other lizards [63,65].

The occurrence of females bearing both oviductal eggs and vitellogenic follicles indicates that *A. ameiva* has the potential to exhibit a continuous cyclic gonadal pattern at the individual level [31]. However, the frequency with which individual females repeat successive reproductive events within the same reproductive season requires further investigation. At the population level, reproductive females concentrate in specific periods of the year in all three populations, thus indicating a seasonal cycle. This cycle is not fully synchronous, as multiple clutches and variation in clutch frequency result in individuals being at different reproductive stages simultaneously. This overlap is also reflected in follicular development, with females bearing similarly sized follicles at different vitellogenic stages. Consequently, the overall pattern is best described as seasonal and semi-synchronous [31].

### 4.3. Mating season

Across populations, mating coincided with periods of active vitellogenesis and presumed ovulation. Yet the timing of vitellogenesis, the distribution of gravid females and the occurrence of sperm storage differed among regions, indicating that local environmental conditions modulate reproductive dynamics and shape cycles that vary in synchrony and duration. In the equatorial population, mating occurred from the late dry season into the early wet season (October to February; figure 6). This period coincides with the peak of vitellogenesis, when follicles reach preovulatory sizes (>10 mm [8]). A similar pattern occurred in the tropical wet–dry population, where mating concentrated between September and March, overlapping with follicular growth and the presumed timing of ovulation (figure 6). In this population, however, stored sperm in the aglandular uterus suggests that these gametes may fertilize a clutch during the dry season (May–July; figure 4*c*) and may also contribute to a subsequent clutch in the wet season (November–February; figure 4*c*). Sperm detected in the infundibulum during the wet season indicates upward migration and supports the idea that sperm storage is an important reproductive tactic that enables multiple clutches and extends reproductive opportunities (figures 4*c* and 6). Mating plugs found in both populations—reported here for the first time in *A. ameiva*—confirm copulatory activity but require further studies to clarify their function. Finally, in the tropical equatorial population, mating occurred from the late wet season through most of the dry season (April–September), encompassing nearly the entire progression of vitellogenesis through ovulation. The presence of gravid females beginning in March further supports this association (figure 6).

**Figure 6.**
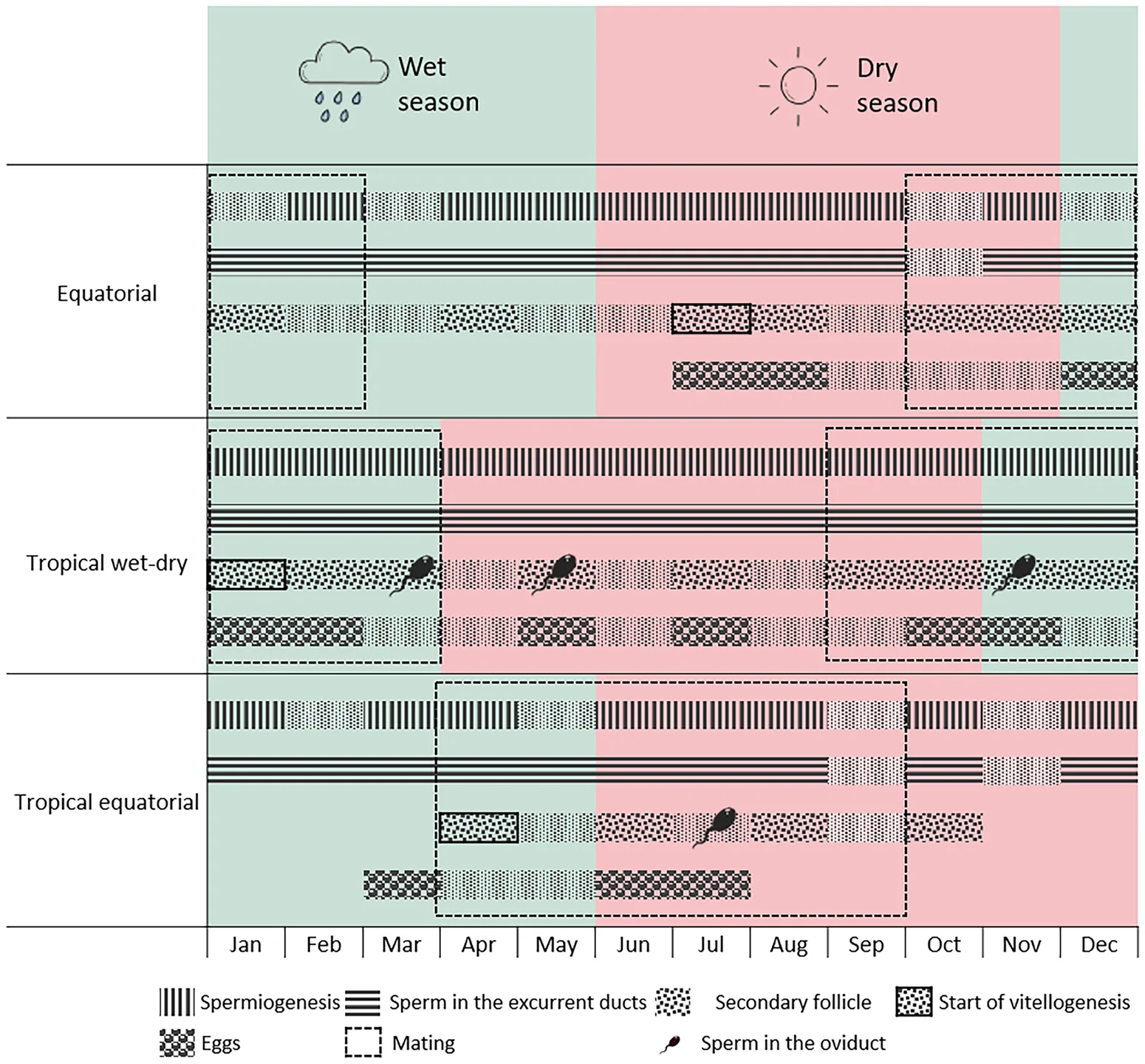
Summary of male and female reproductive events in *A. ameiva* across three Brazilian climatic domains. Dotted segments indicate periods inferred from limited sample availability. Sperm icons denote the presence of sperm within the oviduct.

Taken together, populations inhabiting more predictable climates (equatorial and tropical wet–dry) showed longer and less synchronized vitellogenic periods, indicating greater temporal flexibility. By contrast, reproductive events in the more climatically variable tropical equatorial region were more tightly synchronized. These patterns suggest that climatic predictability shapes the timing and synchrony of female reproductive events in *A. ameiva*.

## 5. Conclusion

We show that *A. ameiva* maintains a continuous cyclical reproductive pattern at the individual level but a seasonal and semi-synchronous pattern at the population level. This combination ensures a year-round supply of gametes and may enable multiple clutches. Differences among populations, especially shifts in the timing of vitellogenesis and mating in the tropical equatorial climate, reinforce the species's reproductive plasticity under contrasting climatic conditions. These temporal adjustments, most evident in females, suggest that local environmental factors shape reproductive dynamics, generating population-specific reproductive cycles. Therefore, our histological analyses suggest that physiological plasticity to environmental variation is an important mechanism supporting the wide geographic distribution of this species.

## Supplementary Material



## Data Availability

The datasets supporting this article have been uploaded as part of the supplementary material [66].
