# Supplementary material for: Reproductive plasticity in the lizard Ameiva ameiva across distinct climatic domains in Brazil
Source: R Soc Open Sci. 2026 Oct 28;13(10):262126. doi: 10.1098/rsos.262126 (PMC13714389; doi:10.1098/rsos.262126)
Supplement: Supplementary file 1 [file rsos.262126.zip › rsos.262126/rsos262126_si_001.docx]

**Table S1.** Voucher numbers of the specimens of *Ameiva ameiva* examined in this study.

| **Population** | **State, location and voucher-ID** |
| --- | --- |
| Equatorial | Acre, Cruzeiro do Sul (ZUEC 202, 212, 213, 355), Mâncio Lima (ZUEC 220, 222), Thaumartugo (ZUEC 1606, 1607); Amazonas, Benjamin Constant (MCN 7528, 7529), Manaus (INPA 11687, 11726, 12039, 18524, 18526, 18527, 19891, 33876, 35431, 35438, 35956, 40958, ZUEC 347, 370, 374, 375), Presidente Figueiredo (INPA 9741, 9743, 9744, 20571, 20575, 20576, 20577, 29478, 30895, 31389, MZUFV 124, 125), RDS Rio Negro (INPA 38986), Santo Antônio do Içá (MCN 7588, 7589), São Paulo de Olivença (MCN 4086); Mato Grosso, Paranaíta (ZUEC 3463). |
| Tropical wet-dry | Bahia, Irecê (ZUEC 350, 351, 363, 364, 365, 366, 367, 368); Espírito Santo, Conceição da Barra (MNRJ 10328, 10330), Linhares (MBML 685, 4424, 4526, ZUEC 1790), Marechal Floriano (MBML 4216), Pedro Canário (ZUEC 361), Praia das Neves (MBML 4141), Presidente Kennedy (MCNR 3842, 4008, MNRJ 18715), Santa Tereza (MBML 509), São Mateus (MBML 136, MNRJ 23982, 23984), Vitória (MNRJ 26382); Goiás, Aporé (MCP 18080), Itajá (ZUFMS 3210), Minaçu (MCP 8303, 12871, 12873, 12874, 12877, 12879, 12920), Mineiros (ZUEC 1961), Paraúna (IBSPCR 2761, 2763); Mato Grosso, Alto Taquari (ZUFMS 1842), Barão de Melgaço (MHNCI 683), Novo São Joaquim (MCP 18019), Poconé (MHNCI 690, 9467); Mato Grosso do Sul, Água Clara (ZUFMS 3362), Corumbá (CEUCH 3622, MNRJ 23187, 23188, ZUFMS 521, 1331, 1332, 2425, 2430, 2431, 2432, 3067, 3206, 3208, 3436), Dois Irmãos do Buriti (CEUCH 2960), Três Lagoas (ZUEC 3503, 3504); Minas Gerais, Aimorés (MCNR 1394, MZUFV 972, 973, 975), Almenara (MZUFV 402), Bonfinópolis de Minas (MZUFV 669), Cabeceira Grande (MCP 19225), Conceição do Mato Dentro (MCNR 6175), Esmeraldas (ZUEC 353), Francisco Sá (MCNR 2135, 2136, 2139), Guarda Mor (MCNR 3173), Grão Mogol (MCNR 1634, 2155, MNRJ 25023, 25110), Inhapim (MZUFV 766), Itabirinha de Mantena (MZUFV 69), João Pinheiro (MZUFV 1446), Pirapora (MNRJ 10489, 10494), Rio Piracicaba (MCNR 5912), Santana do Riacho (ZUEC 520), Tiradentes (CHUFJF 789), UHE Irapé (MCNR 912, 913, 1839, 2257, 2287, 2288, 2981, 2982, 2983, 2997), UHE Miranda (MCRN 790), Várzea da Palma (MZUFV 415, 416); Piauí, Palmeira do Piauí (IBSPCR 353, 380), São Raimundo Nonato (ZUEC 875); Rio de Janeiro, Cabo Frio (MNRJ 15406), Campos dos Goytacazes (MNRJ 10325), Carapebus (MNRJ 11077, 27680, 27681), Duque de Caxias (MNRJ 17011), Iguaba Grande (MNRJ 17538, 17540, 17541), Jacarepaguá (MNRJ 10272), Macaé (MNRJ 24549), Magé (MNRJ 18109), Maricá (MHNCI 1684, MNRJ 13200, 13201, 13205), São Pedro da Aldeia (MNRJ 10319), Tanguá (MNRJ 10273); São Paulo, Araçatuba (ZUEC 2198, 2962), Indaiatuba (ZUEC 354); Tocantins, Caseara (ZUFMS 3374), UHE Peixe Angical (IBSPCR 2159, 2310). |
| Tropical equatorial | Ceará, Aracati (CHUFC 6005, 6006), Barbalha (URCA 1440), Crateús (CHUFC 2798), Crato (URCA 4, 5), Fortaleza (CHUFC 1374, 1606, 3486, 7170, 7171, 7203, 7575), Guaramiranga (CHUFC 3126), Ibiapina (CHUFC 2059, 2730, 2731), Iguatu (CHUFC 1595), Maranguape (CHUFC 5685), Mulungu (CHUFC 1568, 1596, 3123), Nova Olinda (URCA 8349), Pacoti (CHUFC 3120), Paraipaba (URCA 5796), Pecém (CHUFC 3580, 3715, 3716, 3717), São Gonçalo do Amarante (CHUFC 3151, 4116), Serra da Ibiapaba (CHUFC 3050, 3140), Tianguá (CHUFC 2729, 2780), Trairi (CHUFC 7789), Ubajara (ZUEC 3419); Maranhão, Alcântara (ZUEC 3324). |

CEUCH: Universidade Federal de Mato Grosso do Sul, Campus de Corumbá; CHUFC: Universidade Federal do Ceará; CHUFJF: Universidade Federal de Juiz de Fora; IBSPCR: Instituto Butantan; INPA: Instituto Nacional de Pesquisas da Amazônia; MBML: Museu de Biologia “Mello Leitão” do Instituto Nacional da Mata Atlântica; MCN: Museu de Ciências Naturais do Rio Grande do Sul; MCNR: Museu de Ciências Naturais da Pontifícia Universidade Católica de Minas Gerais; MCP: Museu de Ciências e Tecnologia da Pontifícia Universidade Católica do Rio Grande do Sul; MHNCI: Museu de História Natural Capão da Imbuia; MNRJ: Museu Nacional do Rio de Janeiro; MZUFV: Museu de Zoologia “João Moojen”; URCA: Universidade Regional do Cariri; ZUEC: Museu de Biodiversidade da Universidade Estadual de Campinas; ZUFMS: Universidade Federal de Mato Grosso do Sul.

**Table S2.** Results of ANCOVA and Mann Whitney Wilcoxon tests for testicular volume (TV) and ductus deferens diameter (DD) in different populations of *Ameiva ameiva*.

| **Population** | **Trait** | **Predictor** | **Df** | **Statistic** | ***p*-value** |
| --- | --- | --- | --- | --- | --- |
| Equatorial | TV | Season | 1 | F = 2.87 | 0.118 |
|  |  | SVL | 1 | F = 20.51 | < 0.001 |
|  | DD | Season | - | U = 29.00 | 0.957 |
| Tropical wet-dry | TV | Season | 1 | F = 0.007 | 0.930 |
|  |  | SVL | 1 | F = 54.25 | < 0.001 |
|  | DD | Season | 1 | F = 0.09 | 0.763 |
|  |  | SVL | 1 | F = 40.99 | < 0.001 |
| Tropical equatorial | TV | Season | 1 | F = 0.17 | 0.680 |
|  |  | SVL | 1 | F = 17.54 | < 0.001 |
|  | DD | Season | 1 | F = 3.77 | 0.065 |
|  |  | SVL | 1 | F = 1.95 | 0.176 |
